# A Dual-Function Fe-Doped Co_3_O_4_ Nanosheet Array for Efficient OER and HER in an Alkaline Medium

**DOI:** 10.3390/molecules30051046

**Published:** 2025-02-25

**Authors:** Yibo Su, Bo Liu, Zijun Shi, Mei Yan, Tengfei Ma

**Affiliations:** 1 School of Chemistry and Chemical Engineering, Inner Mongolia University of Science & Technology, Baotou 014010, China; 2 Bureau of Science & Technology Talent, Ordos High-Tech Industrial Development Zone, Ordos 017000, China

**Keywords:** electrocatalysis, oxygen evolution reaction (OER), Ni foam, hydrogen evolution reactions (HER), d-band center shift

## Abstract

The electrocatalysts of heteroatom-doped non-precious metal oxide materials are of great significance for efficient and low-cost electrochemical water-splitting systems. Herein, an innovative Fe-doped Co_3_O_4_ nanoflake (Fe-Co_3_O_4_/NF) on nickel foam has been developed, which exhibits excellent electrocatalytic activity for both hydrogen evolution reactions (HERs) and oxygen evolution reactions (OERs). Benefiting from the synergy of the charge redistribution and d-band center shift caused by doping engineering, the as-obtained Fe-Co_3_O_4_/NF shows both excellent HER (*η*_10_ = 196 mV) and OER (*η*_10_ = 290 mV) activities with low Tafel slopes (109 mV dec^−1^ for HER and 49 mV dec^−1^ for OER, respectively) and excellent stability. This work provides an effective method for designing and synthesizing bifunctional electrocatalysts with high activity and stability of metal oxide hybrids for the HER/OER.

## 1. Introduction

With the increasingly severe global energy crisis and environmental pollution problems, finding efficient and clean renewable energy has become an urgent task. Therefore, hydrogen energy has attracted much attention due to its advantages such as high energy density and zero emissions, and is regarded as an important part of the future energy system [1,2,3]. Alkaline water electrolysis for hydrogen production technology, as a mature hydrogen production method, has the characteristics of simple operation, low cost, and environmental friendliness, and occupies an important position in the hydrogen energy industry chain [4,5,6]. However, the traditional alkaline water electrolysis technology has problems such as high energy consumption and low efficiency, which limit its large-scale application. Therefore, the development of efficient and stable electrocatalysts is the key to improving the efficiency of alkaline water electrolysis for hydrogen production [7,8,9].

Nickel foam (NF), as a three-dimensional porous material, has a high specific surface area, good electrical conductivity, and excellent chemical stability, and is an ideal electrode material [10,11,12]. And cobalt oxide (Co_3_O_4_), as a transition metal oxide, shows great potential in the field of electrocatalysis due to its good catalytic activity and low cost [13,14,15]. Through in situ growth technology, Co_3_O_4_ nanostructures can be directly formed on the surface of NF, avoiding the material transfer or composite interface problems that may occur in the traditional preparation methods. This integrated design not only simplifies the preparation process, but also greatly improves the stability and durability of the electrode due to the tight combination between the nanostructure and the substrate [16,17,18,19,20]. In addition, the in situ grown Co_3_O_4_ nanostructures usually have a higher specific surface area and more active sites, which are crucial for improving the catalytic efficiency of water electrolysis for hydrogen production [21].

Although Co_3_O_4_ in situ grown on NF shows good catalytic performance in hydrogen production, stability remains an urgent problem to be solved [22]. After working for a long time, Co_3_O_4_ may fall off or undergo phase transformation, which will result in a decrease or even failure of catalytic activity [23,24,25]. To improve its stability, researchers have adopted various strategies, such as introducing doping elements and optimizing the preparation process [26,27,28]. Pan et al. proposed a strategy of synergistically improving catalytic activity through Er doping to enhance the intrinsic activity of OER and the stability of Co_3_O_4_. At 10 mA cm^−2^, the overpotential of the Er-Co_3_O_4_ catalyst with an Er content of 4% is 321 ± 5 mV, and the stability is significantly improved for more than 250 h [13]. Jiang et al. designed Co-Mn bimetallic nanowires of Co_3_S_4_/Mn_3_O_4_ and Co_2_P/MnP. The interfacial interaction of Co_3_S_4_/Mn_3_O_4_ changes the electronic structure of Co_3_S_4_, partially fills the oxygen vacancies of Mn_3_O_4_, and effectively promotes the stability of OER under alkaline conditions. Adding Mn_3_O_4_ and MnP to modulate Co_3_S_4_ and Co_2_P can reduce the overpotential and extend the stability of the electrolytic water reaction [29]. Except for the problem of stability, another key challenge is the slow reaction kinetics, which mainly includes two aspects, the slow electron transfer rate, and the blocked mass transfer. Researchers proposed methods of optimizing the microstructure of Co_3_O_4_ by adjusting the preparation conditions and introducing the synergistic effect between bimetals through doping heteroatom to solve the problems. Wang et al. used ZIF-67 as the precursor and template to prepare a series of defect-rich N-doped Co_3_O_4_/C porous nano cube catalysts (N-Co_3_O_4_@NC) through a controllable N doping technique. The introduction of N atoms can optimize the electronic structure of Co atoms, resulting in a decrease in the electron density around Co and the formation of N-containing catalytic active sites. Moreover, the N doping and substitution of lattice O in Co_3_O_4_ will inevitably lead to the formation of oxygen vacancies, which will effectively improve the conductivity of the electrocatalyst [30]. It is worth noting that the difference in the radius between the heteroatom and Co atom may cause lattice distortion or lattice defects in Co_3_O_4_, which promotes the redistribution of electrons at the two-phase interface, optimizes the adsorption and transformation of active sites to reaction intermediates, increases the electrical conductivity, and improves the catalytic activity.

In this work, a simple in situ hydrothermal synthesis method was employed to prepare flower-like Fe-Co_3_O_4_/NF as a bifunctional electrocatalyst for HER and OER. Firstly, the flower-like Co_3_O_4_ was in situ grown on the conductive substrate NF, which possesses a high specific surface area and a large number of active sites. Then, the thickness of the Co_3_O_4_ nanosheets was altered by doping of the Fe atom, which is beneficial for improving the structural stability of the composite electrocatalyst. Simultaneously, Fe doping effectively regulates the electronic structure of the active center and promotes the redistribution of charges. Moreover, the upward shift of the d-band center caused by the doping engineering enhances the adsorption ability of the electrocatalyst surface to the reaction radicals, thereby improving the HER and OER activities. Fe-Co_3_O_4_/NF exhibits excellent HER performance (overpotential of 196 mV @ 10 mA cm^−2^) and OER performance (overpotential of 290 mV @ 10 mA cm^−2^) in the 1 M KOH electrolyte solution, and can maintain stability for 200 h in 1 M KOH.

## 2. Results and Discussion

### 2.1. Morphologic and Composition Characterization

**Figure 1:**
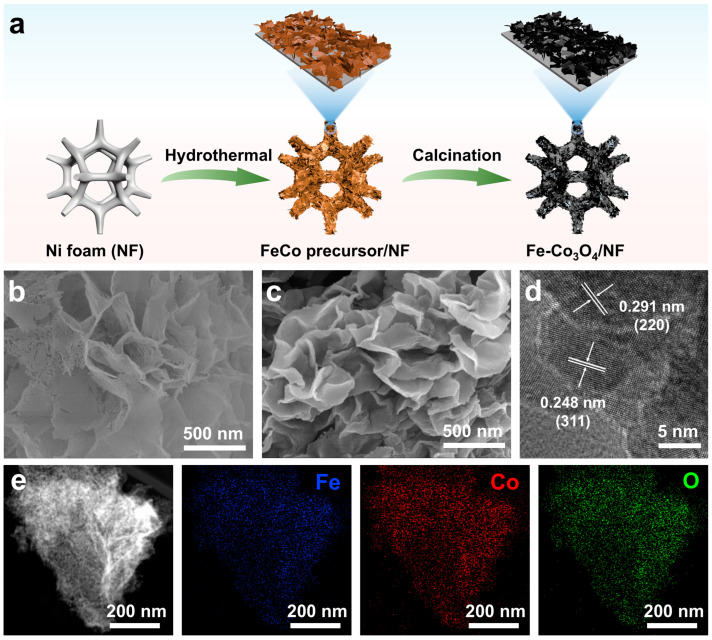
(**a**) Schematic illustration of the synthesis of Fe-Co_3_O_4_/NF. SEM images of (**b**) Co_3_O_4_/NF and (**c**) Fe-Co_3_O_4_/NF. (**d**) TEM image of Fe-Co_3_O_4_/NF. (**e**) The EDS mapping images of Fe, Co, and O.

Fe-Co_3_O_4_/NF-integrated electrocatalyst was synthesized by two steps of a simple hydrothermal reaction and calcination oxidation process (Figure 1a). Firstly, a bimetallic FeCo precursor/NF with a nanosheet morphology was grown on an NF substrate via a simple solvothermal method. Subsequently, the obtained FeCo precursor/NF was calcined in air at 350 °C for 2 h to prepare the final Fe-Co_3_O_4_/NF. For comparison, Co_3_O_4_/NF integrated catalyst was prepared using the same synthesis method just without the Fe precursor.

The microstructure of the samples was analyzed by scanning electron microscopy (SEM) and transmission electron microscopy (TEM). As shown in Figure S1a, Co_3_O_4_/NF exhibits a layered nanoflower structure, and it is further observed from Figure 1b that the lamellar structure is assembled from nanoparticles. After doping with Fe, Fe-Co_3_O_4_/NF still maintains a similar layered nanoflower structure to Co_3_O_4_/NF, but the thickness of the lamellae increases significantly, which is beneficial to improving the stability of the catalyst in the reaction process (Figure S1b and Figure 1c).

It is further confirmed by transmission electron microscopy (TEM) that the lamellar structure in the powder sample obtained by ultrasonication of the prepared Fe-Co_3_O_4_/NF is composed of nanoparticles (Figure 1d), which is consistent with the result of SEM. Further measurement and analysis of its lattice show that the lattice spacings of 0.291 nm and 0.248 nm correspond to the (220) and (311) crystal planes of Co_3_O_4_, respectively. Compared with the corresponding Co_3_O_4_ (0.286 nm and 0.244 nm of (220) and (311), JCPDS No.42-1467), the lattice spacing exhibits a slight expansion after the doping of Fe, which proves the successful introduction of Fe in Fe-Co_3_O_4_/NF. The EDX mapping image in Figure 1e indicates that the Fe, Co, and O elements are uniformly distributed on Fe-Co_3_O_4_, also indicating that the Fe species have been successfully introduced into the sample.

**Figure 2:**
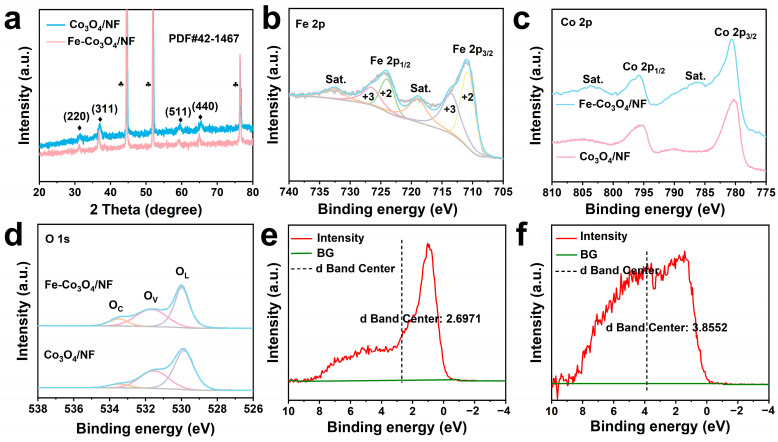
Structural and compositional characterization of the as-prepared samples. (**a**) XRD patterns. High-resolution XPS spectra of (**b**) Fe 2p, (**c**) Co 2p, and (**d**) O 1s. The d-band center position was obtained from the valence-band spectra of (**e**) Co_3_O_4_/NF and (**f**) Fe-Co_3_O_4_/NF.

To investigate the structure of the catalyst, X-ray diffraction (XRD) tests were conducted on the prepared catalysts. As shown in Figure 2a, Fe-Co_3_O_4_/NF has almost the same diffraction peaks as Co_3_O_4_/NF (JCPDS No. 42-1467), mainly corresponding to the (220), (311), (511), and (440) crystal planes of Co_3_O_4_. After doping with Fe species, no characteristic peaks of Fe and its related compounds are formed. However, compared with Co_3_O_4_/NF, the characteristic diffraction peaks of Fe-Co_3_O_4_/NF shift to a small angle, which indicates that the doping of Fe will cause the lattice expansion of Co_3_O_4_ (which consists of the TEM results).

To further explore the changes caused by the doping of Fe, the elemental composition and chemical state of Co_3_O_4_/NF and Fe-Co_3_O_4_/NF were analyzed using XPS. The high-resolution Fe 2p spectrum of Fe-Co_3_O_4_/NF shows two corresponding satellite peaks at 718.89 eV and 732.67 eV (Figure 2a). The fitting peaks at 710.9 eV and 723.4 eV are attributed to Fe^2+^, while 714.1 eV and 726.7 eV are attributed to Fe^3+^, indicating the coexistence of Fe^2+^ and Fe^3+^.

Although XPS cannot directly observe the physical structure of oxygen vacancy, the existence of oxygen vacancy and its related chemical state and electronic structure can be indirectly proved by analyzing the information of peak position, shape, and intensity in XPS spectra. For the spectra of O 1s (Figure 2b), the three peaks from high binding energy to low binding energy correspond to chemisorbed oxygen (O_C_), surface oxygen vacancies (O_V_), and lattice oxygen (O_L_), respectively. The peak area proportions of O_C_, O_V_, and O_L_ of Co_3_O_4_/NF and Fe-Co_3_O_4_/NF are obtained through the peak fitting. As shown in Table S1, the O_V_ concentration increases from 35.95% of Co_3_O_4_/NF to 40.25% of Fe-Co_3_O_4_/NF, indicating the successful entrance of doping Fe in the crystal lattice of Co_3_O_4_, formatting the oxygen vacancies, which facilitates electron conduction and ion transportation.

The Co 2p XPS spectrum of the Co_3_O_4_/NF (Figure 2c) shows two peaks at 780.23 eV and 795.21 eV belonging to Co 2p_3/2_ and Co 2p_1/2_, respectively. Compared with the Co_3_O_4_/NF, the binding energy of Co 2p_3/2_ in Fe-Co_3_O_4_/NF shifts 0.35 eV, indicating that the electron cloud density around Co reduces due to the influence of the doping atom Fe. The introduction of Fe alters the electronic structure of Co_3_O_4_, increases the unpaired electrons, leads to a stronger electron correlation effect, and causes a charge redistribution between Co and O atoms, enhancing the electron–electron interaction, thereby strengthening the satellite peaks.

It is well known that the position of the d-band center is directly related to the adsorption energy of the intermediate. This is because the change in the adsorption energy of various intermediates to different metals determines the activity and stability of the electrocatalyst, so the d-band center is an important indicator of HER and OER performance [31]. The approximate d-band center position can be obtained from the valence-band spectra [32]. As shown in Figure 2e,f, the d-band center of Fe-Co_3_O_4_/NF is approximately 1.16 eV higher than that of Co_3_O_4_/NF. According to the d-band center theory, the increase in the d-band center energy level enhances the adsorption ability of the catalyst surface to the reactive free radicals, which theoretically can improve the activities of HER and OER.

### 2.2. HER Electrocatalytic Performance

**Figure 3:**
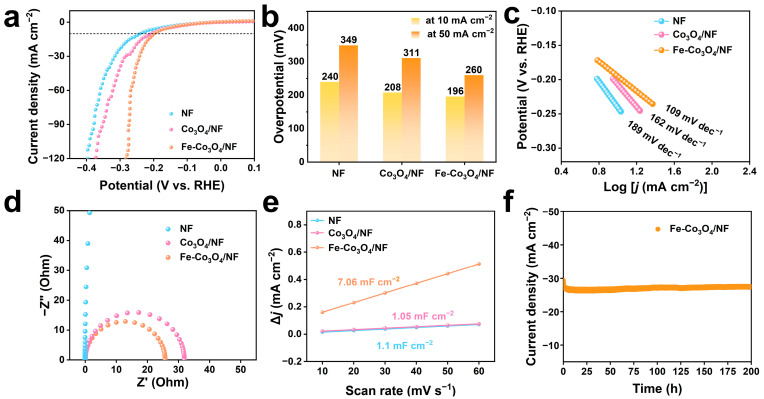
Electrochemical tests of Fe-Co_3_O_4_/NF and other catalysts for HER in 1 M KOH solution. (**a**) The LSV polarization curves at a scan rate of 2 mV s^−1^. (**b**) Overpotential at 10 and 50 mA cm^−2^. (**c**) Tafel plots of various HER electrocatalysts. (**d**) EIS spectra. (**e**) Linear fit of scan rate to capacitive current density of NF, Co_3_O_4_/NF, and Fe-Co_3_O_4_/NF. (**f**) The i-t tests performed for various electrocatalysts at the current density of 27.5 mA cm^−2^.

The electrocatalytic HER performance of NF, Co_3_O_4_/NF, and Fe-Co_3_O_4_/NF was tested in a 1.0 M KOH electrolyte using a typical three-electrode system. As presented in Figure 3a,b, polarization curves are obtained by LSV with a scanning rate of 2 mV s^−1^. The Fe-Co_3_O_4_/NF shows a remarkable catalytic HER activity with a much lower η_10_ of only 196 mV than Co_3_O_4_/NF (η_10_ = 208 mV) and bare NF (η_10_ = 240 mV). It is worth noting that at 50 mA cm^−2^ high current density, the overpotential required for Fe-Co_3_O_4_/NF is also lower than that for Co_3_O_4_ and NF. Moreover, the Fe-Co_3_O_4_/NF exhibits favorable HER kinetics with a Tafel slope of 109 mV dec^−1^, which is smaller than that of Co_3_O_4_ (162 mV dec^−1^) and NF (189 mV dec^−1^) (Figure 3c). When the electrode reaction is strongly affected by the diffusion process, such as when the diffusion rate of reactants or products near the electrode surface is slow and cannot be supplemented or removed in time, it will lead to the deviation of the relationship between the current density and the electrode potential from the ideal situation and increase the Tafel slope, which even may exceed 120 mv/dec. EIS measurements reflect the reaction kinetics and conductivity of the catalyst in the HER process, and the fitted Nyquist diagram is shown in Figure 3d. Fe-Co_3_O_4_/NF exhibits the smallest charge transfer resistance, demonstrating its desirable reaction kinetics and excellent electrical conductivity. In addition, CV curves for the non-faraday region (Figure S2) are collected to determine the C_dl_ (Figure 3e, relative to the RHE). The Fe-Co_3_O_4_/NF possesses a C_dl_ of 7.06 mF cm^−2^, significantly higher than those of Co_3_O_4_/NF (1.05 mF cm^−2^) and NF (1.1 mF cm^−2^), indicating the exposure of more active sites. Moreover, the stability of the catalyst is also important for practical applications. The durability of the electrocatalysts was assessed using current–time (i–t) chronoamperometric measurement. As shown in Figure 3f, the current density of Fe-Co_3_O_4_/NF at 27.5 mA cm^−2^ exhibits a negligible change during continuous operation over 200 h. The polarization curves also show the same result before and after the 1000-cycle voltammetry cycles (Figure S3).

### 2.3. OER Electrocatalytic Performance

**Figure 4:**
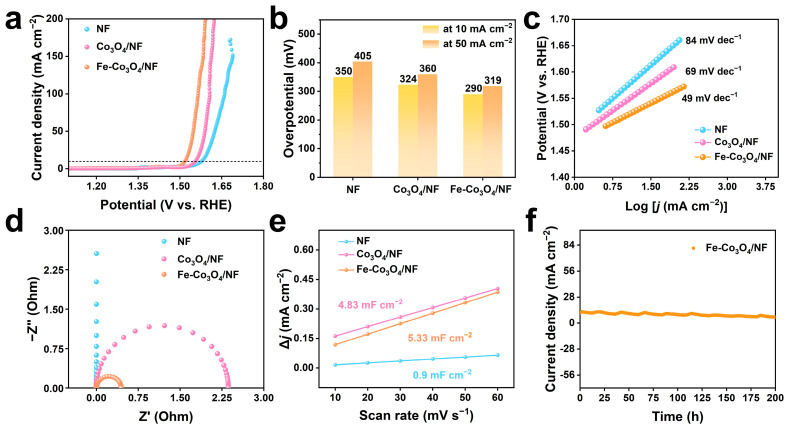
Electrochemical tests of Fe-Co_3_O_4_/NF and other catalysts for OER in 1 M KOH solution. (**a**) The LSV polarization curves at a scan rate of 2 mV. (**b**) Overpotential at 10 and 50 mA cm^−2^. (**c**) Tafel plots of various OER electrocatalysts. (**d**) EIS spectra. (**e**) Linear fit of scan rate to capacitive current density of NF, Co_3_O_4_/NF, and Fe-Co_3_O_4_/NF. (**f**) The i-t tests performed for various electrocatalysts at the current density of 10 mA cm^−2^.

The OER performances of NF, Co_3_O_4_/NF, and Fe-Co_3_O_4_/NF catalysts were further investigated under the same test conditions as above. Figure 4a shows the polarization curves of different catalysts with a potential scanning rate of 2 mV s^−1^. As shown in Figure 4b, the as-prepared Fe-Co_3_O_4_/NF exhibits outstanding OER activity and only requires an overpotential of 290 mV at 10 mA cm^−2^ and 319 mV at 50 mA cm^−2^, which is superior to those of Co_3_O_4_/NF (324 mV and 360 mV) and NF (350 mV and 405 mV). The Fe-Co_3_O_4_/NF also shows a much smaller Tafel slope (49 mV dec^−1^) than those of Co_3_O_4_/NF (69 mV dec^−1^) and NF (84 mV dec^−1^), suggesting it has excellent electrochemical water oxidation kinetics (Figure 4c). The Nyquist radius of Fe-Co_3_O_4_/NF exhibited the lowest impedance compared to the other catalysts, which was favorable for the OER process (Figure 4d). Moreover, CV curves for the non-faraday region (Figure S3) were collected to determine the double-layer capacitance (C_dl_). Figure 4e shows that the C_dl_ of Fe-Co_3_O_4_/NF, Co_3_O_4_/NF, and NF are estimated as 5.33 mF cm^−2^, 4.83 mF cm^−2^, and 0.9 mF cm^−2^, respectively. The changing trend of the C_dl_ value is like the activity sequence, indicating that the electronic structure is significantly optimized after Fe doping, thus achieving enhanced adsorption/desorption performance. The difference in C_dl_ values between HER and OER can be explained by two factors. Firstly, in HER, fewer intermediates and simpler adsorption/desorption make electrode surface double-layer structure changes less complex. In OER, intermediate adsorption and reaction alter charge distribution and electric field, causing more obvious double-layer changes. Secondly, HER has moderate hydrogen atom adsorption energy and quick gas generation/desorption, keeping a stable double layer. active catalysts of OER form oxygen-rich layers, enhancing charge storage and increasing capacitance. These jointly lead to C_dl_ value differences. In addition, the durability of Fe-Co_3_O_4_/NF was further assessed at a fixed current density of 10 mA cm^−2^ (Figure 4f). the Fe-Co_3_O_4_/NF current density decreased by only 5.6% after 200 h of operation, showing excellent durability. Moreover, the polarization curves of Fe-Co_3_O_4_/NF before and after 1000 CV cycles remained almost invariant (Figure S4), implying a stable OER activity. In addition, the SEM of the catalyst after HER/OER was characterized (Figure S5), and it can be found that the morphology of the sample has slight changes after testing.

In order to explore the role of 2-methylimidazole, the ligand-free samples were prepared and the HER and OER properties were tested. As shown in Figure S6, for HER performance, the sample without ligand preparation is significantly inferior to the previous Fe-Co_3_O_4_/NF. As for the OER performance, it is also inferior to the sample with a ligand at a higher current density. Therefore, 2-methylimidazole as a ligand coordinates with cobalt ions to form cobalt-based derivatives with unique structures and properties after high-temperature calcination, which is conducive to improving the activity of the catalyst, especially for HER.

To further investigate the influence of Fe salt content on the catalytic performance, in addition to the sample with 0.2 g of iron salt, samples with iron salt contents of 0.1 g and 0.3 g (denoted as 0.1 Fe-Co_3_O_4_/NF and 0.3 Fe-Co_3_O_4_/NF, respectively) were prepared, and their catalytic performances were evaluated. As shown in Figure S7, the HER activity of 0.2 Fe-Co_3_O_4_/NF is significantly superior to that of 0.1 Fe-Co_3_O_4_/NF and 0.3 Fe-Co_3_O_4_/NF, while the OER performance of all three samples is nearly identical. Therefore, 0.2 Fe-Co_3_O_4_/NF is identified as a more effective catalyst.

## 3. Materials and Methods

### 3.1. Materials

Cobalt nitrate hexahydrate (Co(NO_3_)_2_·6H_2_O, AR, 99%), ferric nitrate nonahydrate (Fe(NO_3_)_3_·9H_2_O, AR, 98.5%), 2-methylimidazole (2-mim, C_4_H_6_N_2_, 98%), and potassium hydroxide (KOH, 95%) were purchased from Macklin Biochemical Technology Co., Ltd., Shanghai, China. Acetone (CH_3_COCH_3_, AR) and hydrochloric acid (HCl, AR) were purchased from Kelong Chemical Co., Ltd., Chengdu, China. Methanol anhydrous (CH_3_OH, AR) and ethanol (C_2_H_5_OH, AR) were obtained from Kemiou Chemical Reagent Co., Ltd., Tianjin, China. All the reagents and solvents were used without further purification. Nickel foam (NF, 100 × 100 × 1.0 mm) was purchased from Suzhou Astronergy New Material Co., Ltd., Suzhou, China. Deionized (DI) water was homemade.

### 3.2. Synthesis of Materials

Co_3_O_4_/NF was in situ synthesized on NF by the hydrothermal method. Typically, a commercial NF was cut into rectangular pieces (3 × 3 cm^2^), and, respectively, washed with acetone, and diluted HCl (3 M) for 5 min to remove impurities. Then, the NF was rinsed with ethanol and DI water several times and dried to obtain the cleaned NF for further use.

Firstly, 1.02 g of Co(NO_3_)_2_·6H_2_O was dissolved in 15 mL of methanol to form a solution A, and 0.616 g of 2-methylimidazole was dissolved in 15 mL of methanol to form a solution B. Then, a mixture of solution A and solution B was transferred into a 50 mL Teflon-lined stainless-steel autoclave with a piece of vertically inserted cleaned NF and kept at 120 °C for 4 h. After naturally cooling to room temperature, the sample was collected and washed several times with ethanol and water and dried in a vacuum oven at 60 °C for 6 h. The sample was placed in a muffle furnace and calcined in air at 350 °C for 2 h to obtain Co_3_O_4_/NF.

Firstly, 1.02 g of Co(NO_3_)_2_·6H_2_O and 0.202 g of Fe(NO_3_)_3_·9H_2_O were dissolved in 15 mL of methanol to form a solution A, 0.616 g of 2-methylimidazole was dissolved in 15 mL of methanol to form a solution B. Then, a mixture of solution A and solution B was transferred into a 50 mL Teflon-lined stainless-steel autoclave with a piece of vertically inserted cleaned NF and kept at 120 °C for 4 h. After natural cooling to room temperature, the sample was collected and washed several times with ethanol and water, and dried in a vacuum oven at 60 °C for 6 h. Finally, the sample was placed in a muffle furnace and calcined in air at 350 °C for 2 h and was labeled as Fe-Co_3_O_4_/NF.

### 3.3. Characterization

The structural and compositional information of Co_3_O_4_/NF and Fe-Co_3_O_4_/NF were detected by scanning electron microscope (SEM, Zeiss 360 Sigma, Oberkochen, Germany), transmission electron microscopy, high-resolution electron microscopy, and energy-dispersive X-ray spectroscopy mapping with an acceleration voltage of 200 kV (TEM, HRTEM, and EDS-Mapping, JEM-2800, JEOL, Tokyo, Japan). The material microstructure of the samples was examined by X-ray diffractometer (XRD, Rigaku Ultima IV, Tokyo, Japan) from 10° to 90° with a speed of 10° min^−1^. X-ray photoelectron spectroscopy measurement was used to analyze the surface chemical states of the samples (XPS, ESCALAB 250Xi, Al Kα, Thermo Scientific, Waltham, MA, USA).

### 3.4. Electrochemical Measurements

Electrochemical tests were performed on the CorrTest electrochemical workstation. The HER and OER properties of the catalyst were tested with a three-electrode system 1 M KOH electrolyte. The samples were directly used as the working electrode, while the graphite rod and Hg/HgO electrode served as counter electrode and reference electrode, respectively. All potential values were calibrated with a reversible hydrogen electrode (RHE) according to the Nernst equation: E_RHE_ = E_Hg/HgO_ + 0.0592 × pH + 0.098. The working electrode was cycled at a scan rate of 50 mV s^−1^ for 30 cycles to activate the electrode in the electrolyte. Linear sweep voltammetry (LSV) measurements were obtained at a sweep rate of 2 mV s^−1^ with 90% iR compensation. The electrical double layer capacitance (C_dl_) was calculated from cyclic voltammograms (CV) obtained in the non-Faraday interval at scanning rates of 10, 20, 30, 40, 50, and 60 mV s^−1^. The ECSA can be obtained by the formula ECSA = C_dl_/C_s_. Electrochemical impedance spectroscopy (EIS) was tested in the frequency range of 100 kHz to 0.01 Hz and an AC amplitude of 5 mV. The long-term stability test was measured by chronopotentiometry at a fixed current density of 27.5 mA cm^−2^ (HER) and 10 mA cm^−2^ (OER) for 200 h.

## 4. Conclusions

In summary, the introduction of Fe heteroatoms into Co_3_O_4_ by doping engineering was reported to enhance the activity of the HER and OER in alkaline conditions. Impressively, the Fe-Co_3_O_4_/NF require a low potential of 196 mV to achieve a current density of 10 mA cm^−2^ for the HER and 290 mV to achieve 10 mA cm^−2^ for the OER. The Fe-Co_3_O_4_/NF towards alkaline HER and OER all exhibited high stability and can maintain a current density of 27.5 mA cm^−2^ and 10 mA cm^−2^ for 200 h, respectively. The experimental results indicated that the essential factor in promoting electrocatalytic performance is due to the charge redistribution and the upward shift of the d-band center caused by Fe doping. This study is of significance for the design and development of highly efficient and stable HER and OER bifunctional electrocatalysts in alkaline media.

## Data Availability

The data presented in this study are available in the article.

## Supplementary Materials

The following supporting information can be downloaded at https://www.mdpi.com/article/10.3390/molecules30051046/s1, Figure S1: the photos of NF, FeCo precursor/NF and Fe-Co_3_O_4_/NF; Figure S2: SEM images of (a) Co_3_O_4_/NF and (b) Fe-Co_3_O_4_/NF; Figure S3: HER CV curves of (a) NF, (b) Co_3_O_4_/NF, (c) Fe-Co_3_O_4_/NF with different scan rates from 10 to 60 mV s^−1^, and (d) polarization curves of Fe-Co_3_O_4_/NF electrode before and after 1000 CV cycles in 1.0 M KOH; Figure S4: OER CV curves of (a) NF, (b) Co_3_O_4_/NF, (c) Fe-Co_3_O_4_/NF with different scan rates from 10 to 60 mV s^−1^, and (d) polarization curves of Fe-Co_3_O_4_/NF electrode before and after 1000 CV cycles in 1.0 M KOH; Figure S5: SEM images of Fe-Co_3_O_4_/NF after (a) HER and (b) OER; Figure S6: the LSV polarization curves of Fe-Co_3_O_4_/NF with and without 2-methylimidazole at a scan rate of 2 mV s^−1^ for (a) HER and (b) OER; Figure S7: the LSV polarization curves of Fe-Co_3_O_4_/NF with different amount of Fe for (a) HER and (b) OER. Table S1: the calculated OC, OV, and OL proportion from XPS spectra of Co_3_O_4_/NF and Fe-Co_3_O_4_/NF.
