# Supplementary material for: A Dual-Function Fe-Doped Co3O4 Nanosheet Array for Efficient OER and HER in an Alkaline Medium
Source: Molecules. 2025 Feb 25;30(5):1046. doi: 10.3390/molecules30051046 (PMC11902015; doi:10.3390/molecules30051046)
Supplement: Supplementary file 1 [file molecules-30-01046-s001.zip › molecules-3454690-supplementary.pdf]

Supporting Information

A dual-functional Fe-doped Co_3O_4 nanosheet array for efficient OER and HER in an alkaline medium

Yibo Su,[a] Bo Liu,[a] Zijun Shi,[b] Mei Yan,[a] Tengfei Ma[a,*]

[a] School of Chemistry and Chemical Engineering, Inner Mongolia University of Science & Technology, Baotou 014010, China
[b] Bureau of Science & Technology Talent, Ordos High-Tech Industrial Development Zone, Ordos 017000, China
E-mail: matf@mail.nankai.edu.cn

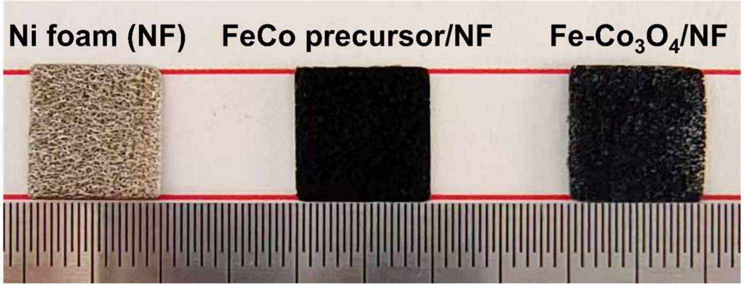

Figure S1. The photos of NF, FeCo precursor/NF and Fe-Co_3O_4/NF.

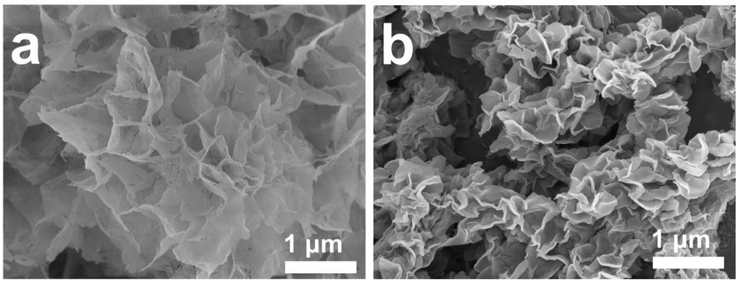

Figure S2. SEM images of (a) Co_3O_4/NF and (b) Fe-Co_3O_4/NF.

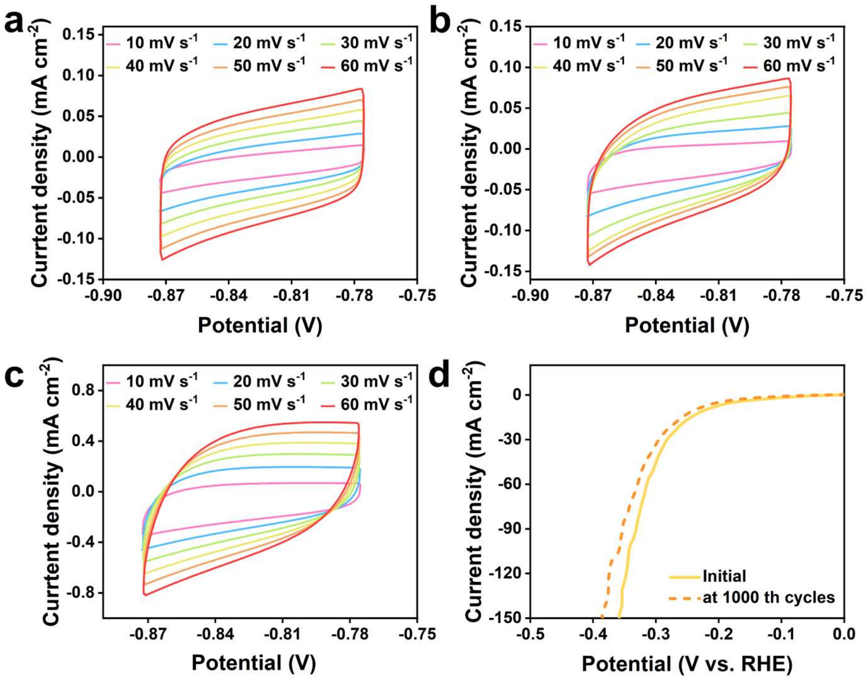

Figure S3. HER CV curves of (a) NF, (b) Co_3O_4/NF and (c) Fe-Co_3O_4/NF with different scan rates from 10 to 60 mV s^{-1}. (d) Polarization curves of Fe-Co_3O_4/NF electrode before and after 1000 CV cycles in 1.0 M KOH.

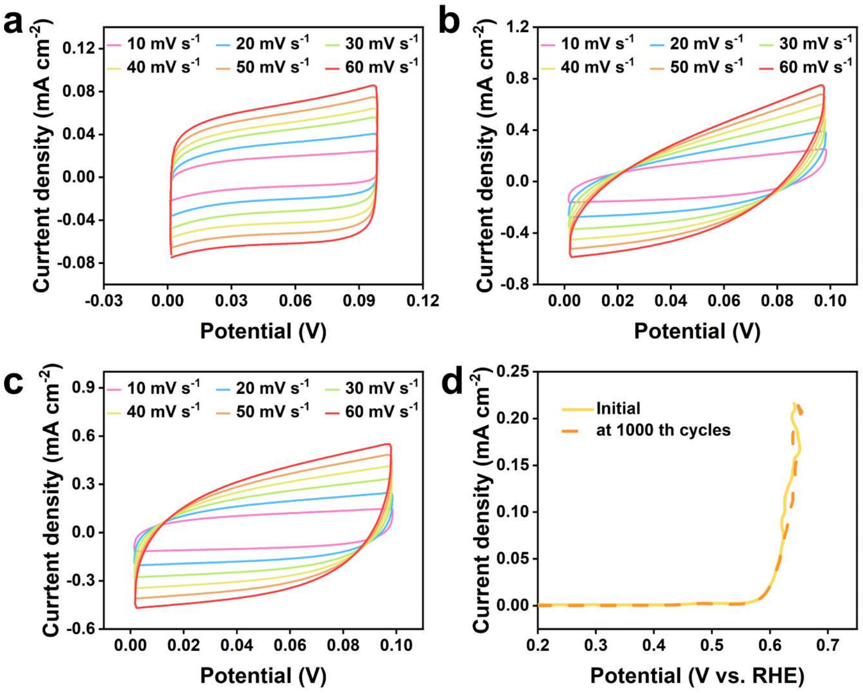

Figure S4. OER CV curves of (a) NF, (b) Co_3O_4/NF and (c) Fe-Co_3O_4/NF with different scan rates from 10 to 60 mV s^{-1}. (d) Polarization curves of Fe-Co_3O_4/NF electrode before and after 1000 CV cycles in 1.0 M KOH.

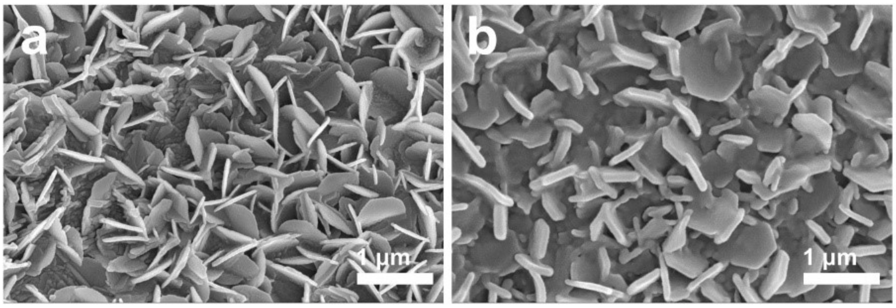

Figure S5. SEM images of Fe-Co_3O_4/NF after (a) HER and (b) OER.

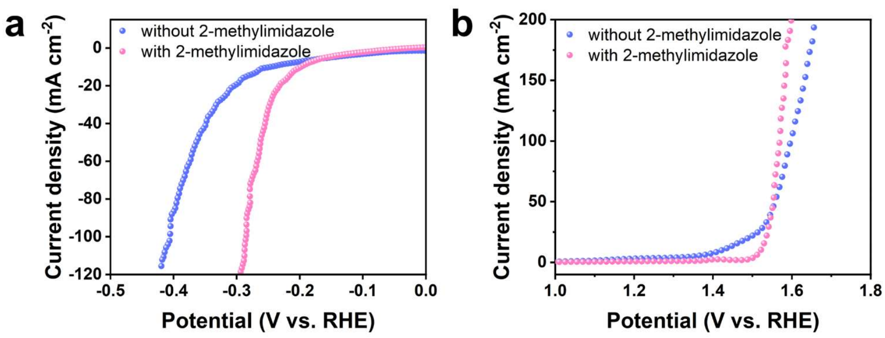

Figure S6. The LSV polarization curves of Fe-Co_3O_4/NF with and without 2-methylimidazole at a scan rate of 2 mV s^{-1} for (a) HER and (b) OER.

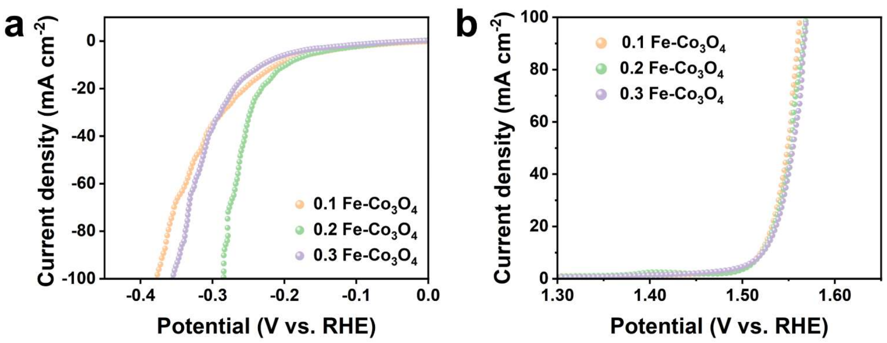

Figure S7. The LSV polarization curves of Fe-Co_3O_4/NF with different amount of Fe for (a) HER and (b) OER.

Table S1. The Calculated O_C、O_V、O_L proportion from XPS spectra of Co_3O_4/NF and Fe-Co_3O_4/NF

Proportion (%)	Co_3O_4/NF	Fe-Co_3O_4/NF
O_C	7.13	9.42
O_V	35.95	40.25
O_L	56.92	50.33
